# Novel approach of high cell density recombinant bioprocess development: Optimisation and scale-up from microlitre to pilot scales while maintaining the fed-batch cultivation mode of *E. coli *cultures

**DOI:** 10.1186/1475-2859-9-35

**Published:** 2010-05-20

**Authors:** Juozas Šiurkus, Johanna Panula-Perälä, Uwe Horn, Mario Kraft, Renata Rimšeliene, Peter Neubauer

**Affiliations:** 1 Laboratory of Bioprocess Engineering, Department of Biotechnology, Technische Universität Berlin, Ackerstr. 71-76, D-13355 Berlin, Germany; 2 Fermentas UAB, V. Graiciuno 8, LT-02241 Vilnius, Lithuania; 3 Bioprocess Engineering Laboratory, Department of Process and Environmental Engineering and Biocenter Oulu, University of Oulu, PO Box 4300, FI-90014 Oulu, Finland; 4 Leibnitz Institute for Natural Product Research and Infection Biology, Beutenbergstr. 11a, D-07745 Jena, Germany

## Abstract

**Background:**

Bioprocess development of recombinant proteins is time consuming and laborious as many factors influence the accumulation of the product in the soluble and active form. Currently, in most cases the developmental line is characterised by a screening stage which is performed under batch conditions followed by the development of the fed-batch process. Performing the screening already under fed-batch conditions would limit the amount of work and guarantee that the selected favoured conditions also work in the production scale.

**Results:**

Here, for the first time, high throughput multifactorial screening of a cloning library is combined with the fed-batch technique in 96-well plates, and a strategy is directly derived for scaling to bioreactor scale. At the example of a difficult to express protein, an RNase inhibitor, it is demonstrated that screening of various vector constructs and growth conditions can be performed in a coherent line by (i) applying a vector library with promoters and ribosome binding sites of different strength and various fusion partners together with (ii) an early stage use of the fed-batch technology. It is shown that the EnBase^® ^technology provides an easy solution for controlled cultivation conditions in the microwell scale. Additionally the high cell densities obtained provide material for various analyses from the small culture volumes. Crucial factors for a high yield of the target protein in the actual case were (i) the fusion partner, (ii) the use of of a mineral salt medium together with the fed-batch technique, and (iii) the preinduction growth rate. Finally, it is shown that the favorable conditions selected in the microwell plate and shake flask scales also work in the bioreactor.

**Conclusions:**

Cultivation media and culture conditions have a major impact on the success of a screening procedure. Therefore the application of controlled cultivation conditions is pivotal. The consequent use of fed-batch conditons from the first screening phase not only shortens the developmental line by guarantying that the selected conditions are relevant for the scale up, but in our case also standard batch cultures failed to select the right clone or conditions at all.

## Background

Recombinant production of proteins in heterologous hosts is today one of the key technologies to obtain protein for structural studies, functional characterisation and industrial production. The growing demand for a new therapeutic and commercial recombinant proteins provoked a rapid development of a large number of commercially available prokaryotic and eukaryotic expression systems and hosts.

Generally it is the aim to get the target protein within a short time in sufficient amounts and quality, i.e. in the native folded state. Although successful for a large number of proteins, often the overexpression of recombinant genes in the common host *Escherichia coli *results in aggregation [1], or even no product is accumulated due to degradation. Despite the availability of a large number of host strains, cloning vectors, fusion and coexpression partners, the optimisation procedure may be long-lasting and highly dependent on the experiences of the performing scientist or team.

Recommendations for "first to try" strategies based on the experience from tens of thousands of proteins [2] may be a good guide, however, unfortunately, such strategies often fail for secreted or more complex proteins. In this case often step by step optimisation is performed, starting either with shake flasks or with multiwell plates. The latter ones provide the possibility to evaluate in parallel a large number of conditions (see e.g. [3]). Recent developments provide even the option for on line monitoring of cultivation parameters such as pH, oxygen, or cell density [4-8].

Product yield and degree of aggregation depend on a number of factors which are highly interconnected. Strength of transcriptional induction and ribosome binding efficiency are strongly related to environmental parameters, such as the actual medium composition, the external pH and the growth temperature which also effect the specific growth rate and, overflow metabolism, i.e. acetate production. Environmental conditions rapidly change in shaken batch systems due to the exponential biomass increase and the inability control these factors, which provides variation between cultures; a problem which has been extensively discussed [2].

Furthermore, results from multiwell plate of shake flask optimisation studies are often not directly transferable to bioreactor scale cultures and industrial production, where the aim is to boost the product yield per liter [9]. The standard technology in a bioreactor to increase the cell density and thus the volumetric product yield is the fed-batch technology which is based on a strict control of the environmental conditions and much slower growth rates compared to batch cultures. To avoid repeated optimisation in different cultivation formats, the fed-batch technology should be applied already in the first multiwell scale cultivation platforms, which is not a trivial task. Recently, new innovations paved the way by applying substrate autodelivery solutions [10,11] or by the use of robot based feeding [12].

Here, for the first time, high throughput multifactorial screening of a cloning library is combined with the fed-batch type of cultivation in 96-well plates, and a strategy is proposed for direct scaling to the bioreactor scale. As an example we choose a "difficult to express" eukaryotic protein, an RNase inhibitor (RI) (50 kDa), which strongly aggregates if expressed in *E. coli*. The systematic multifactorial evaluation/screening approach of the gene-dependent genetic factors for protein expression in micro-scale was performed with a library with 45 vectors for cytoplasmic expression, which was constructed by further extending the periplasmic expression vector library from Kraft et al. [7]. The new cytoplasmic expression vector library includes five well known fusion partners and the screening strategy makes use of a luciferase based protein folding reporter system [8]. High throughput, parallel screening in 96 microwell plates for optimal expression followed by evaluation of cultivation factors in shake-flask scale was performed by using the EnBase^® ^fed-batch cultivation technique, and the optimal process was reproduced in 10 L stirred bioreactor cultivations. The experimental results demonstrate a clear advantage for applying the fed-batch technology already during initial screening. Also they prove the straight forward transferability of microwell plate results to the bioreactor scale.

## Results

### Design of the screening vector library

**Figure 1:**
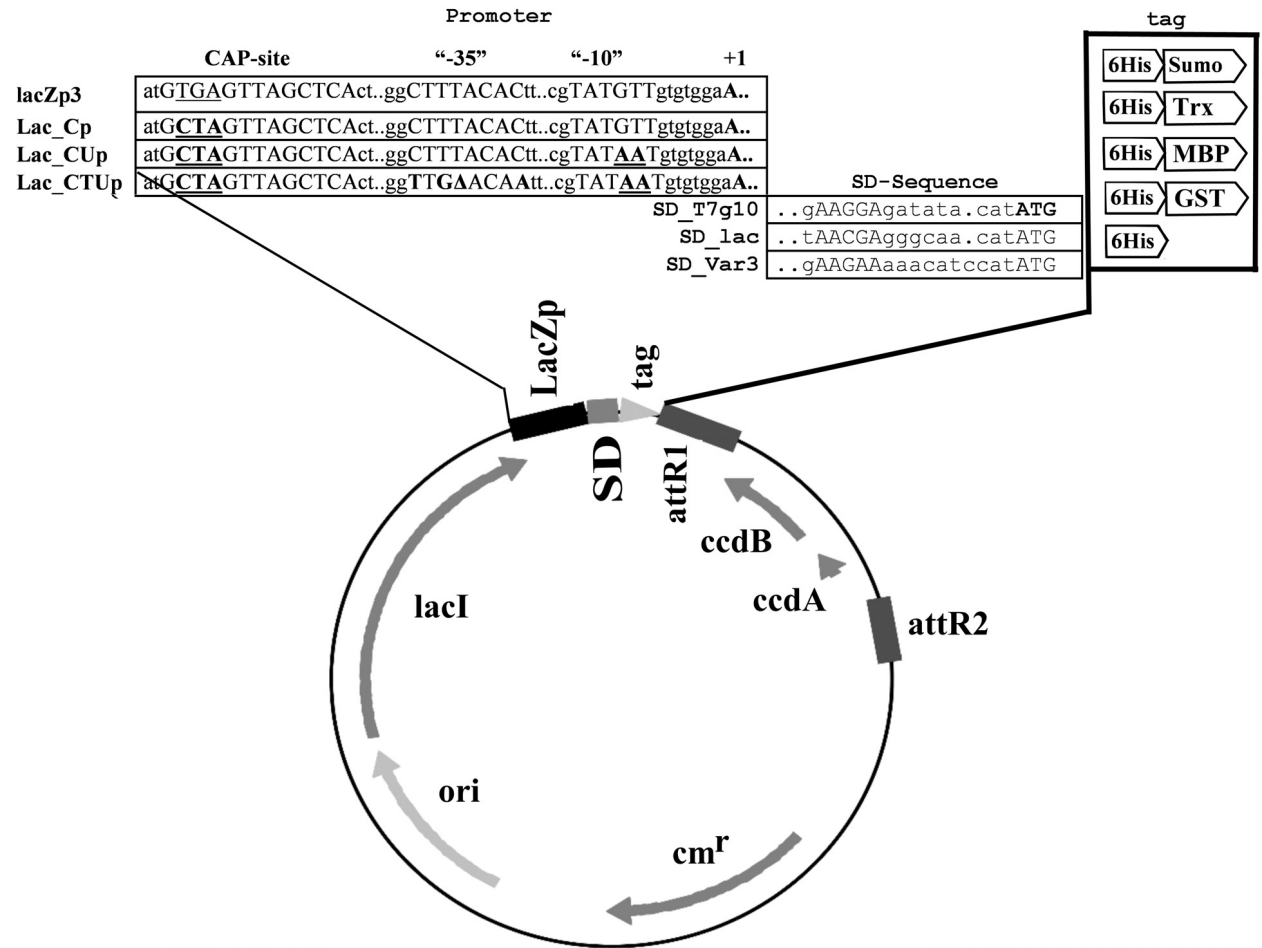
**Schematic presentation of main expression vector library elements**. The library is composed of a plasmid set of 45 vectors based on the pDEST15 vector (Invitrogen) which all contain the ColE1 origin of replication and the chloramphenicol resistance gene (*cm*^*r*^) and the lacI repressor gene. The different plasmids have different *lacZp3*-derived promoters, which were generated by introducing single nucleotide mutations in the "-35" and "-10" regions (bold and underlined letters), and different ribosome binding sites. The promoter and ribosome binding site nomenclatures have been described in detail earlier [6]. In addition the expression vector library contains following target protein N-terminus tag combinations: 6 × His tag, 6 × His-GST (glutathione S-transferase), 6 × His-MBP (maltose-binding protein), 6 × His-SUMO (small ubiquitin-related modifier) and 6 × His-Trx (thioredoxin). The plasmid set is a full factorial combination of the different expression factors.

The cytoplasmic expression vector library based on the expression vector pAK100 [13] and the destination vector pDest15 (Invitrogen, Karlsruhe, Germany) allows a site specific recombination of target genes *via *the *λ *phage attachment sites *att*RI and *att*RII by Gateway cloning. For the preparation of the vector library we used the plasmid vector pAK100 and two further derivates, which contain different variants (Lac_Cp, Lac_Cup, Lac_CTUp) of the native *lac*-promoter providing different levels of transcriptional strength (see figure 1). To reduce the background activity before induction all promoter variants contain in the upstream region the strong transcriptional terminator tHP and additionally a mutation in the CAP-site [13]. The pAK100 derivates where combined *via *PCR with three different ribosome-binding sites (RBS): RBS_*lac *(Genbank accession no. J01636), RBS_T7g10 (Genbank accession no. NC_001604), and a synthetic ribosome binding site (RBS_Var3, [14]). The construction of these parts in the plasmid set was earlier described in detail as a basis for the constructon of a set of plasmids for periplasmic expression [7].

In difference to the earlier set of plasmids, our constructs contained different cytoplasmic fusion partners upstream to the target gene (fused to the N-terminus of the protein), either a simple 6 × His tag, or a 6 × His tag fused with one of the following fusion partners: 6 × His-SUMO (small ubiquitin-related modifier) [15], 6 × His-Trx (thioredoxin), 6 × His-MBP (maltose binding protein MalE), 6 × His-GST (glutathion-S-transferase). Figure 1 shows the schematic summary of the combination of the functional units of the vector set with 45 different plasmids.

### Screening in 96 microwell plates

At the screening stage the whole expression library containing all 45 vectors was cultivated in a 96 microwell plate by the EnBase^® ^technology in 150 μL of MSM. Initially the cultures were started directly in Mineral Salt Medium (MSM) from the glycerol stocks. These cultures showed very high variation in cell densities (data not shown), a subject which was recently extensively discussed by Huber et al. [16].

**Figure 2:**
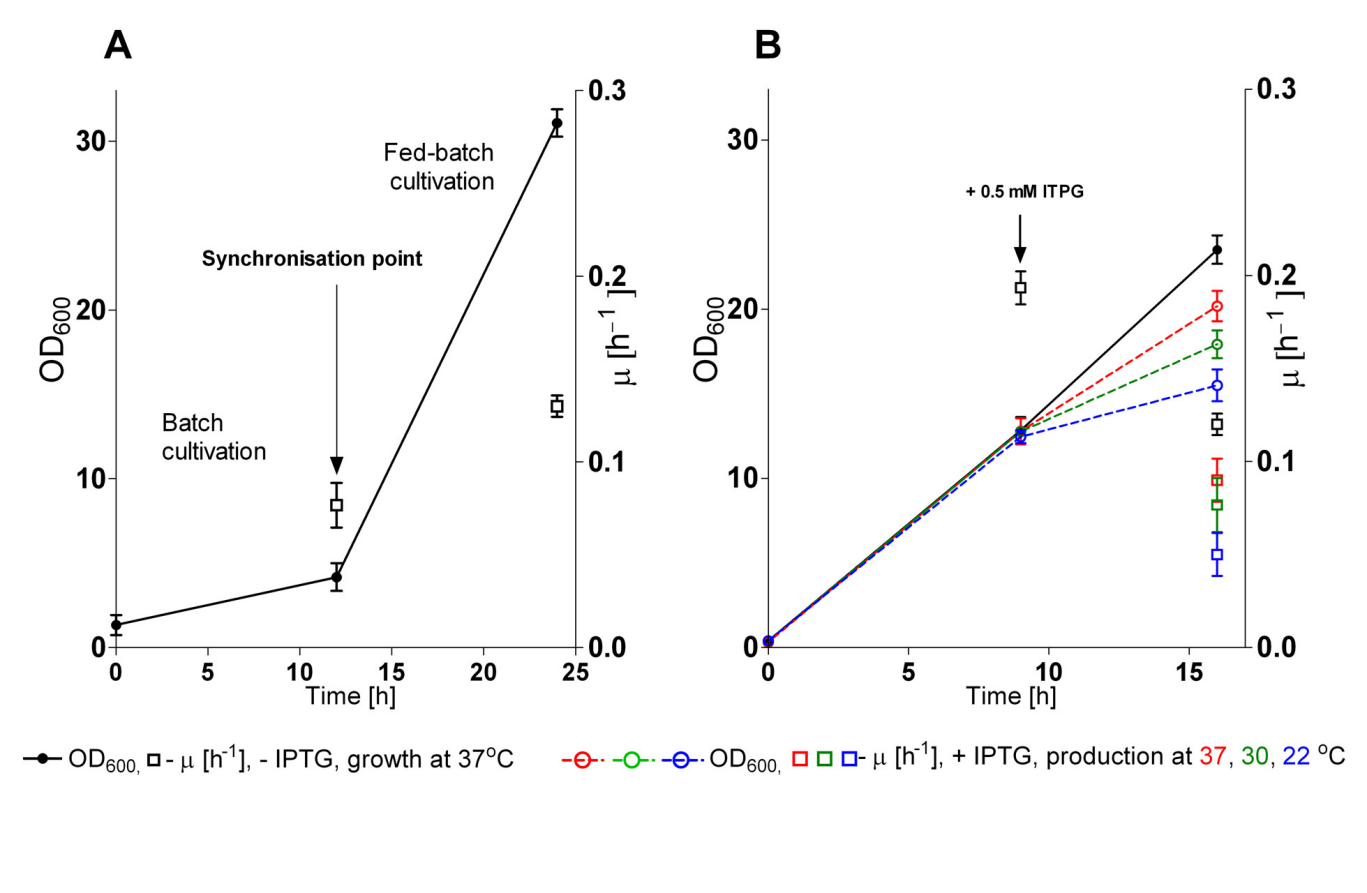
**Growth of the RI expression library consisting of 45 different expression vectors propagated in *E. coli *RV309 p*ibpfxsT7lucA *in 96 microwell plates by the EnBase^® ^technology**. (A) Inocculum preparation and (B) 96 microwell plate expression cultures with plates which were non-induced (closed circle) or set to different postinduction temperatures at the time of induction (37°C - red, 30°C - green, 22°C - blue). Bars indicate the SD of all cultures of a whole plate (including all different vectors). OD_600 _- circles, specific growth rate - squares (average specific growth rate in the time period before).

The problem of variation was solved in our case by equalising the cultures by introducing a first "culture activation stage" directly in the gel-containing EnBase^® ^microwell plates for 12 hours. Therefore in the first step no enzyme was added, but the culture was performed in a typical batch mode with 2.5 g L^-1 ^of glucose (figure 2A). The OD_600 _values of the cultures reached 4.5 ± 1 within 12 hours. At this "synchronization point" glucoamylase (6 AGU L^-1^) was added and thus the continuous release of glucose from the starch substrate was initiated. The enzyme controlled growth-limiting release of glucose maintained synchronized growth of the cultures to similar cell densities for further 12 hours. As a result of this simple synchronization procedure, highly similar growth patterns of all starter cultures were obtained (figure 2A) with an OD_600 _of 31 after 24 hours. This cell material was used as an inocculum for the second expression culture to which the inducer IPTG was added at an OD_600 _of 12 ± 1.0 (figure 2B). At this time all cultures had approximately the same growth rate representing a highly similar physiological state, which was important for evaluation of the parallel screening results for selection of the optimal protein expression construct.

**Figure 3:**
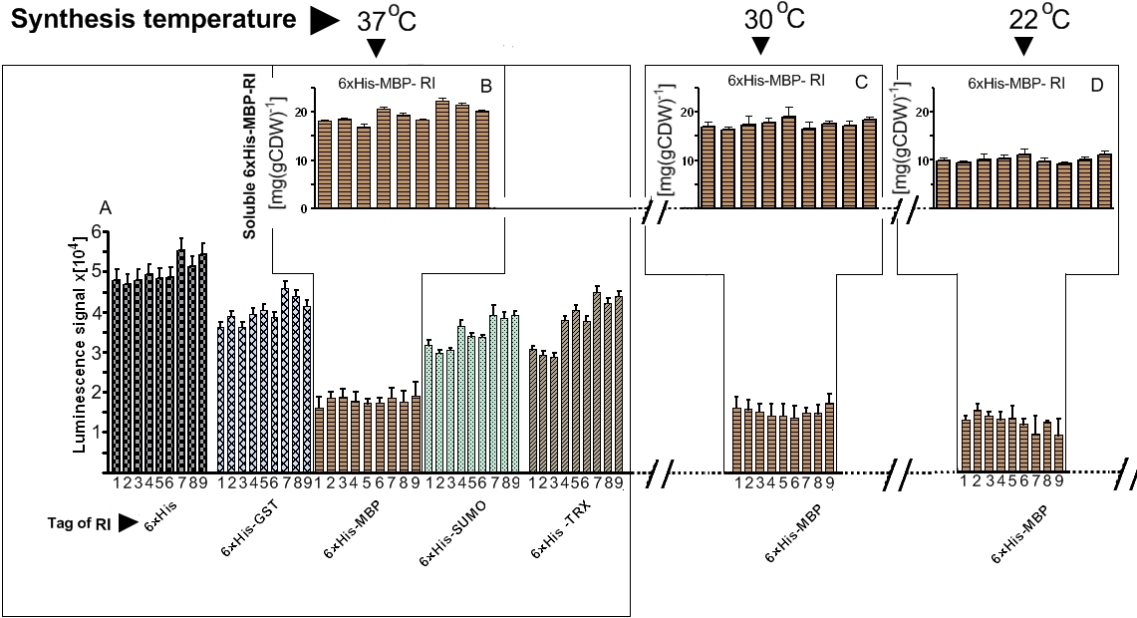
**Aggregation signal measured as luminescence in samples of the RI expression library consisting of 45 different expression vectors propagated in *E. coli *RV309 p*ibpfxsT7lucA *in 96 microwell plates by the EnBase^® ^technology 7 hours after induction**. The lower graph (A) represents luminescence values, reflecting tagged RI protein misfolding stress levels of all expression systems 7 hours after induction with 0.5 mM IPTG. The luminescence values generated by overexpressed luciferase are derived from luciferase activity measurement from cultures performed at 37°C (full set of values displayed) fragments of results derived from cultures performed at 30 and 22°C, for which only the soluble fusion protein giving expression platforms are presented. The bars are numbered in respect to the expression system: 1 - pCT7, 2 - pClac, 3 - pCVar, 4 - pCUT7, 5 - pCUlac, 6 - pCUvar, 7-pCTUT7, 8-pCTUlac, 9-CTUVar. The upper graphs (B, C and D represent soluble fusion protein amounts in mg per gram of cell dry weight [mg (gCDW)^-1^], of the cultures with the 6 × His-MBP-RI fusion constructs at 37°C 30°C and 22°C which gave the lowest luminescence signal.

In this first screening approach the amount of tagged RI fusions produced at either 37, 30, or 22°C in all recombinant clones was monitored at seven hours after addition of the inducer (figures 2B and 3).

For the evaluation of cytoplasmic accumulation of soluble tagged RI, a luminescence based robust recombinant protein folding stress reporter assay was used which was described earlier [8]. Therefore all strains contained, aside from the specific expression plasmid, also the plasmid p*ibpfxs*T7*lucA*, containing the luciferase gene under control of an *E. coli *σ^32^-dependent tandem promoter system derived from the promoters of the *ibpAB *and *fxsA *genes [8]. As shown in the earlier paper, luciferase is strongly expressed when aggregation, i.e. inclusion body formation, occurs in the cell. In our case the measured luminescence signals were highest in the expression strain groups with the expression vectors in which the RI was fused with the tags of 6 × His, 6 × His-GST, 6 × His-Sumo, or 6 × His-Trx (figure 3A for 37°C, Additional file 1 for 30 and 22°C), implying that the product was aggregated in the cultures of these constructs. The relative luminescence between the different strains was similar in the cultures performed at 30 and 22°C compared to the cultures performed at 37°C. However, the lower luminescence values in the 22°C cultures were not due to a lower level of aggregation, but due to the lower level of expression (figure 3D and Additional file 1 part B). Interestingly, no obvious dependency of the aggregation signal at different expression temperatures on the vector promoters and/or strengths of the ribosomal binding site could be observed. Only the group of expression clones in which RI was tagged with the 6 × His-MBP fusion showed a significant lower luciferase activity independently from the construct or the production temperature, i.e. it showed lower aggregation compared to the others (figure 3, Additional file 1). The strains containing the vectors for producing 6 × His-MBP-RI fusion protein had in average a two- to threefold lower luminescence signal compared to the highest signals which was measured from the constructs which only contained the 6 × His-tag at all expression temperatures. These results indicate that MBP serves as a solubilising factor, but interestingly the soluble state is not influenced by the lower temperature or strength of synthesis (figure 3A, Additional file 1).

**Figure 4:**
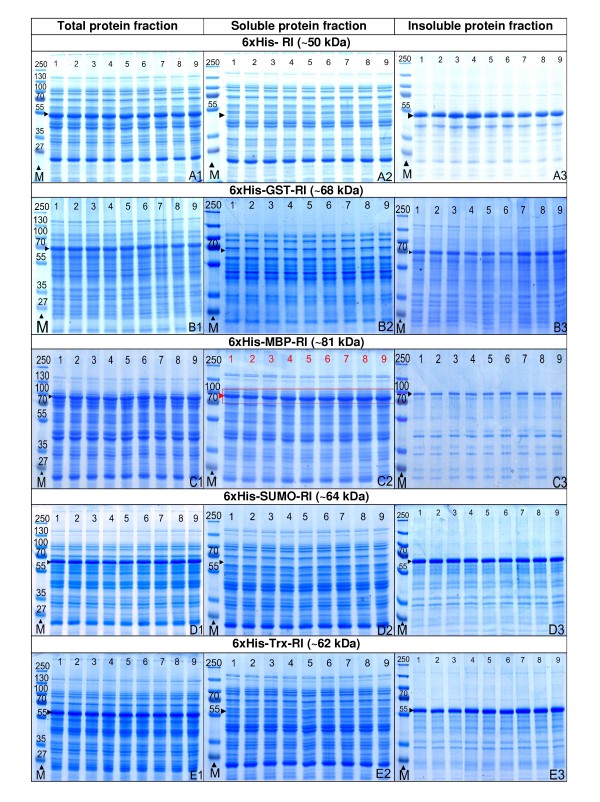
**SDS - PAGE images of total, soluble and insoluble cellular protein fractions normalized to biomass quantity of the 7 h samples of 96 microwell plate cultures of the RI expression library consisting of 45 different expression vectors propagated in *E. coli *RV309 p*ibpfxsT7lucA *by the EnBase^® ^technology performed at 37°C**. The gel lanes are numbered in respect to the expression system: 1 - pCT7, 2 - pClac, 3 - pCVar, 4 - pCUT7, 5 - pCUlac, 6 - pCUvar, 7-pCTUT7, 8-pCTUlac, 9-pCTUVar. Rows: RI produced in total soluble and insoluble protein fractions, tagged with the 6 × His tag (size of fusion protein 50 kDa) (A1-A3); 6 × His-GST (68 kDa) (B1-B3); 6 × His-MBP (81 kDa) (C1-C3); 6 × His-SUMO (64 kDa) (D1-D3); 6 × His-Trx (62 kDa) (E1-E3). Lanes marked with letter M show the protein size marker PageRuler™ Protein Ladder Plus from Fermentas Ltd.

The data derived from the luciferase assay were confirmed by SDS-PAGE analysis of total, soluble, insoluble protein fractions and revealed that the tagged RI was accumulated only in the soluble fraction of the clones with the 6 × His-MBP tag (Fig. 4, SDS-PAGE gel images - C1, C2 and C3). Agilent "LabChip" analysis, used for quantification of 6 × His-MBP-RI in soluble fractions, showed highly similar amounts of soluble target protein for all 6 × His-MBP-RI constructs, independently from the strength of the respective promoter or ribosomal binding site (figure 3B). As there was no obvious disadvantage of using a strong ribosome binding site, and as the strength of induction at the level of transcription later could be easily optimized by varying the amount of inducer, the expression clone containing the strongest promoter (pCTU), the T7 ribosomal binding site, and the 6 × His-MBP fusion partner was selected for further optimisation in larger cultivation scales.

### Conditional screening in shake flasks

After selection of the expression construct which provided a good production of soluble 6 × His-MBP-RI protein in the microwell plate scale, the next question was whether the growth rate at the time of induction would have a major influence on the soluble expression of the target protein.

**Figure 5:**
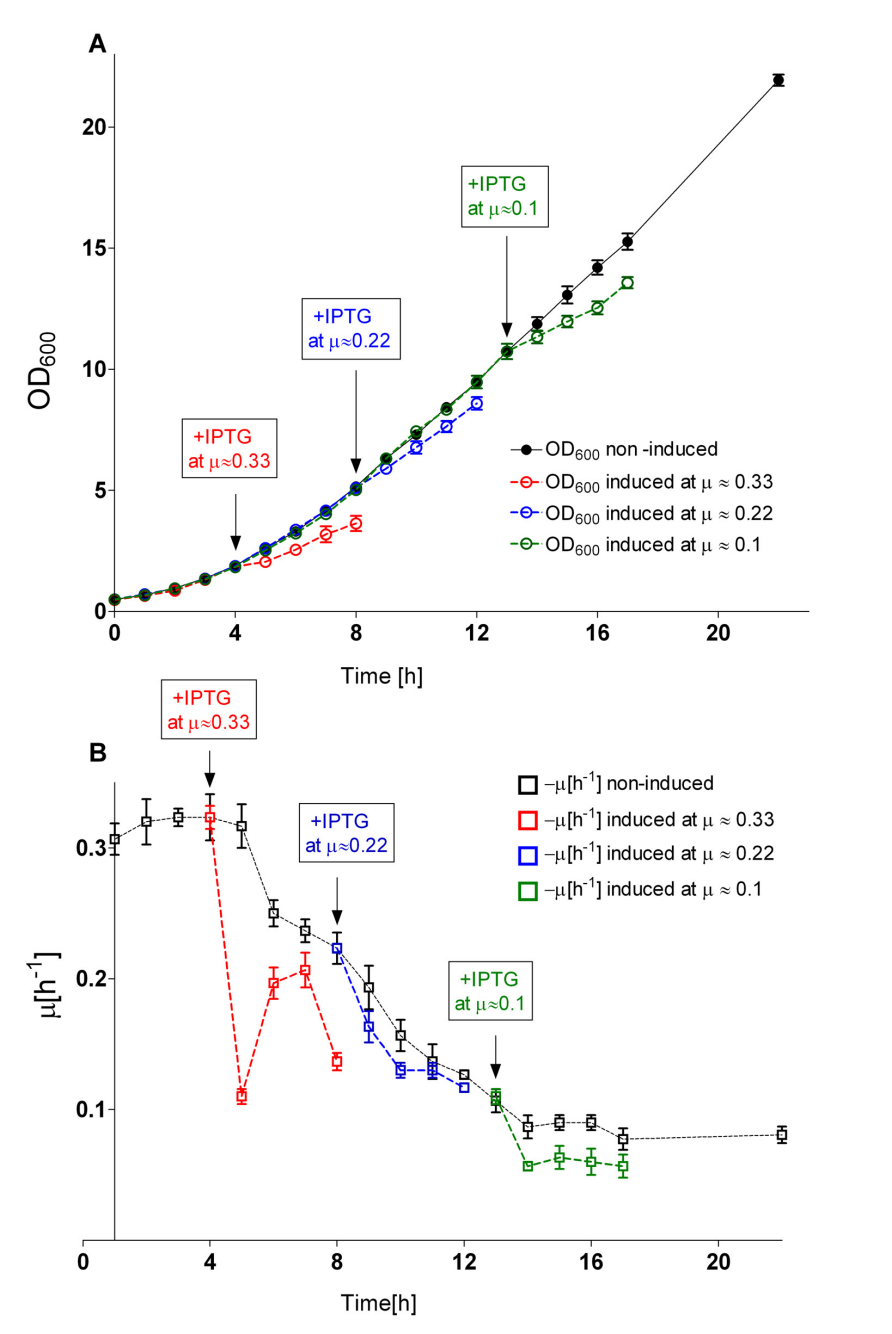
**Shake flask fed-batch culture growth of *E. coli *RV308/p*ibpfxsT7lucA*/pCTUT7MBP-RI with and without target protein production**. (A) Growth curves (OD_600_) of independent fed-batch cultivations at 37°C and the calculated corresponding specific growth rate μ (B). Induction of 6xHis-MBP-RI synthesis was performed at the selected time points when the specific growth rate was about 0.33 h^-1^, 0.22 h^-1^, or 0.1 h^-1^, respectively. The culture cultivation curves were generated from three independent cultivation experiments.

This influence of the specific growth rate was investigated by EnBase^® ^cultivation in 1 L shake flasks with 200 mL of cultivation volume at 37°C. In order to collect reliable data for evaluation of culture behavior during the fed-batch mode in the shake flasks, several fed-batch cultivations with the EnBase^® ^system-generated constant feeding mode were performed under strictly the same cultivations conditions. Due to the high reproducibility of shake flask fed-batch processes, it was possible to reproduce the steadily decreasing specific growth rate from the several, independent cultivation experiments (figure 5). On the basis of derived cultivation data, protein induction was performed at different time points, which each represent a different specific growth rate: μ_1(t1 = 4 h) _≈ 0.33 h^-1^, μ_2(t2 = 7 h) _≈ 0.22 h^-1^, and μ_3__(t3 = 13 h) _≈ 0.1 h^-1 ^(Fig. 5A and 5B). In parallel, as a control, the same expression strain was cultured and induced under batch cultivation conditions in (i) standard LB medium, in 10 g L^-1 ^of glucose containing (ii) semi synthetic and in (iii) MSM medium. In the batch cultures the target protein production was induced at OD_600 _of 1.0 at the maximum specific growth rates. The recombinant production in the cultures cultivated in LB and semi synthetic medium was induced at μ of 0.7 h^-1^, and in glucose-MSM medium at μ of 0.38 h^-1 ^respectively. The target protein synthesis was followed for 4 hours at 37°C in analogy to the EnBase^® ^cultures.

**Figure 6:**
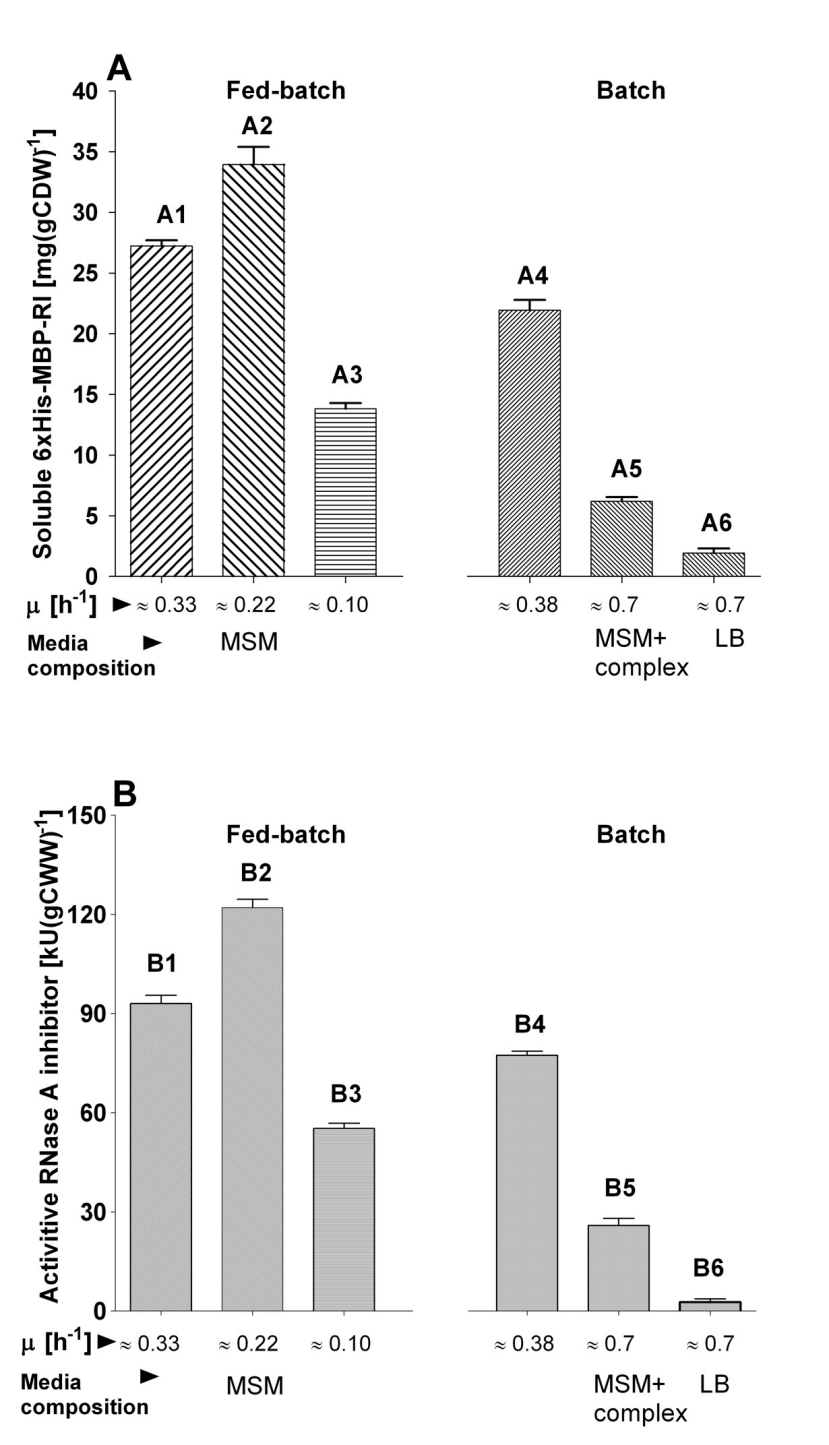
**Chart A: amounts of soluble 6 × His-MBP-RI protein in mg per gram of cell dry weight (mg gCDW^-1^) after shake flask batch and fed-batch cultivation with 6 × His-MBP-RI protein production at 37°C in *E. coli *RV308/p*ibpfxsT7lucA*/pCTUT7MBP-RI**. Columns A1, A2 and A3 represent soluble 6 × His-MBP-RI protein amounts [mg (gCDW)^-1 ^] obtained after 4 hours of fed-batch production at 37°C when induction was performed at different specific growth rates (bars A1-A3). Bars A4-A6 represent the amounts of soluble 6 × His-MBP-RI protein [mg (g CDW)^-1^] after 4 hours of batch production at 37°C in MSM medium containing 10 g L^-1 ^of glucose (column A4), semi-synthetic medium containing 10 g L^-1 ^of glucose (column A5) or LB medium (column A6). Chart B: ribonuclase inhibitor activities (in kilo-units), detected in the cellular crude extracts, calculated for 1 gram of cell wet weight [kU (gCWW)^-1^] after shake flask batch and fed-batch cultivation with 6 × His-MBP-RI protein production at 37°C in *E. coli *RV308/p*ibpfxsT7lucA*/pCTUT7MBP-RI. Columns B1, B2 and B3 represent ribonuclase inhibitor activities obtained after 4 hours of fed-batch production at 37°C when induction was carried at different specific growth rates (bars B1-B3). Bars B4-B4 show ribonuclase inhibitor activities after 4 hours of 6 × His-MBP-RI protein batch production at 37°C in MSM medium containing 10 g L^-1 ^of glucose (column B4), semi-synthetic medium containing 10 g L^-1 ^of glucose (column B5) or LB medium (column B6).

**Figure 7:**
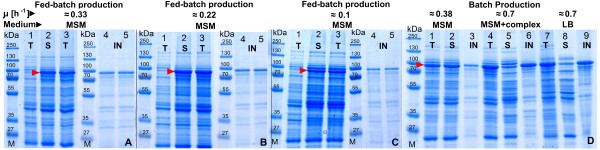
**SDS - PAGE images of different cellular protein fractions after EnBase^® ^fed-batch cultivation of *E. coli *RV308/p*ibpfxsT7lucA*/pCTUT7MBP-RI in shake flasks at 37°C with induction by 0.5 mM IPTG induced at different specific growth rates (A-C) and during batch production with MSM, semi synthetic medium or LB (D)**. Lane abbreviations: (T) - total, (S) - soluble (IN) insoluble protein fractions. Numbered SDS-PAGE gel lanes represent: 1 - total protein fraction 10 min before induction, 2 - soluble protein fraction 4 h after induction, 3 - total protein fraction 4 h after induction. Lanes 4 and 5 - insoluble protein fractions 2 and 4 h after induction. The SDS - PAGE image D represents cellular protein fractions 4 h after batch 6 × His-MBP-RI protein production when induction was carried in batch cultures of MSM medium at OD_600 _= 1. Numbered SDS-Page gel lanes represent protein fractions 4 h after induction: 1, 4 and 7 - total; 2, 5 and 8 - soluble; 3, 6 and 9 - insoluble protein fractions. The gel lanes marked with M represent the protein size marker PageRuler™ Protein Ladder Plus from Fermentas Ltd.

The results indicate a clear impact of the specific growth rate and the cultivation medium or technique on the overall yield of the 6 × His-MBP-RI product (figures 6 and 7). Interestingly, the ratio between soluble and insoluble product was rather constant under all cultivation conditions. The highest amount of soluble 6 × His-MBP-RI protein per cell unit was found in the EnBase^® ^culture induced at a specific growth rate of 0.22 h^-1^, which was slightly higher compared to to induction at a μ of 0.33 h^-1 ^(15% lower yield) and compared to the batch culture on MSM (32% lower), (see figures 6 and 7). A significantly lower product yield (55%) was detected when the culture was induced at μ of 0.1 h^-1^. Interestingly the yield of soluble product was lowest in the cultures which contained complex additives, i.g. in LB and semi-synthetic medium (see figures 6 and 7).

Besides evaluation of 6 × His-MBP-RI soluble protein amounts by using the LabChip approach, also RnaseA inhibitor activities were determined in the normalised crude extracts of biomasses (method according to [17]) produced in previously described fed-batch and batch synthesis experiments (figure 6, chart B). The detected ribonuclease inhibitor activities correlated with the determined soluble 6 × His-MBP-RI amounts and proved the ability of the 6 × His-MBP-RI fusion protein to inhibit Rnase A.

The results from the batch cultivation mode experiments in comparison to the EnBase^® ^cultures indicated, unexpectedly, a lower yield of soluble 6 × His-MBP-RI protein yield per cell unit in all batch cultures (see figures 6 and 7), indicating the importance of the composition of the cultivation medium and the process control scheme. In case of our target protein it was absolutely necessary to perform the whole screening procedure with the fed-batch strategy.

### Scale-up to fed-batch and batch bioreactor cultivations

**Figure 8:**
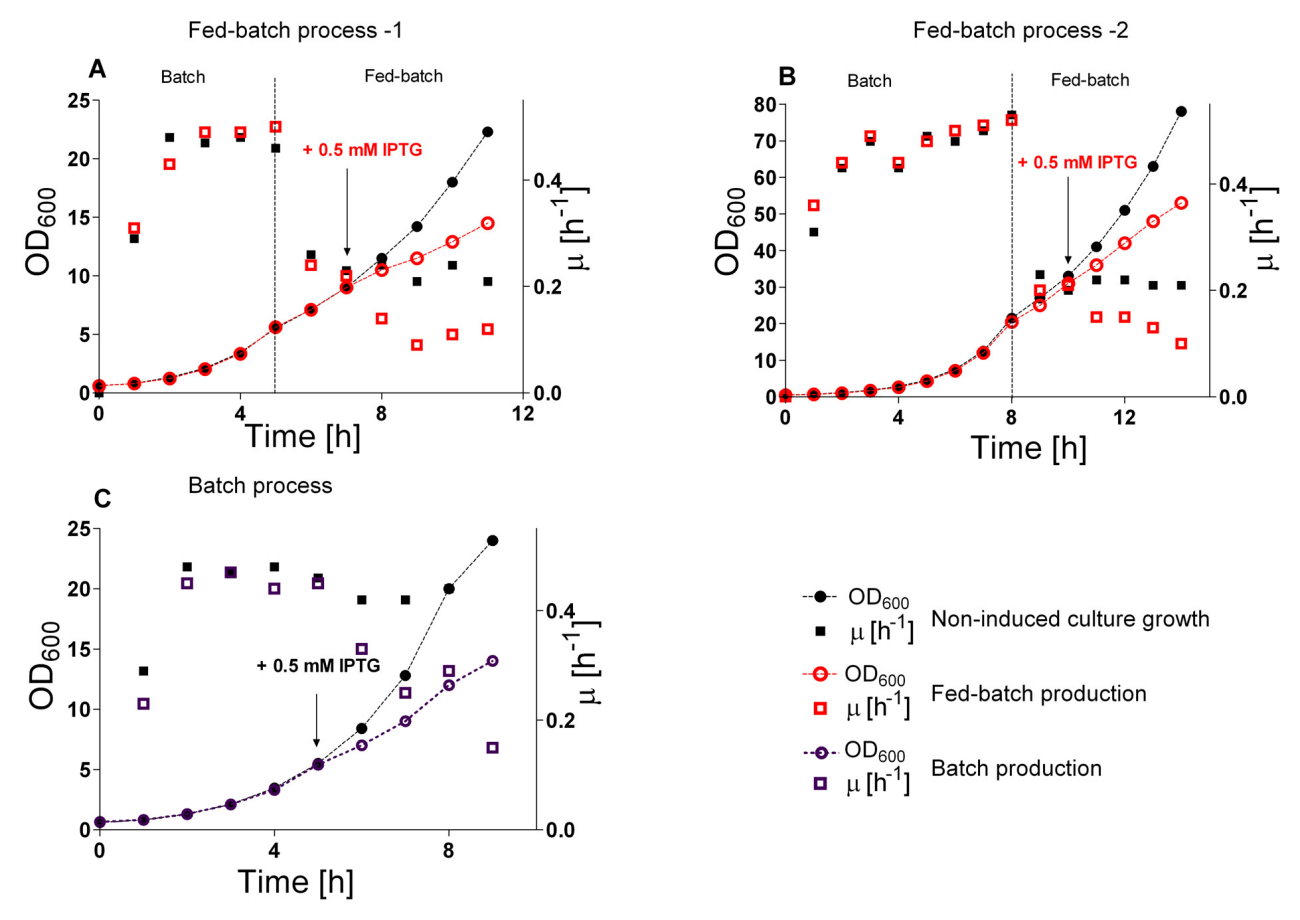
**Growth curves of *E. coli *RV308/p*ibpfxsT7lucA*/pCTUT7MBP-RI cultures during the 6 × His-MBP-RI protein production process in a fed-batch bioreactor with exponential glucose feeding (A and B) and in a batch culture (C). Cultivations were performed at 37°C and induction was performed with 1 M IPTG**. (A) Bioreactor process 1: fed-batch operation was started at OD_600 _= 6, induction at OD_600 _= 9 after the specific growth rate reached μ = 0.22 h^-1^. (B) Bioreactor process 2; fed-batch operation was started at OD_600 _= 22, induction at OD_600 _= 31 after the specific growth rate reached μ = 0.22 h^-1^. (C) Growth curve of the batch bioreactor process. Induction was performed at OD_600 _≈ 5 at a specific growth rate of μ ≈ 0.45 h^-1^.

Finally, as the last optimisation step, optimal 6 × His-MBP-RI protein synthesis was successfully reproduced in the 10 L bioreactor scale at different cell densities (figure 8). In order to create optimal cultivation conditions for the 6 × His-MBP-RI protein induction and production in the bioreactor, a glucose-limited fed-batch process with an exponential feeding profile to guarantee a specific growth rate μ_set _of 0.22 h^-1 ^was applied, according to the best results obtained in the EnBase^® ^shake flask experiments. During the exponential growth phase these fed-batch cultures were induced at a OD_600 _of 9 or 31 (referred to as fed-batch process 1 and 2) and the bioreactor was harvested at 4 hours after induction at OD_600 _of 14 or 50, respectively.

**Figure 9:**
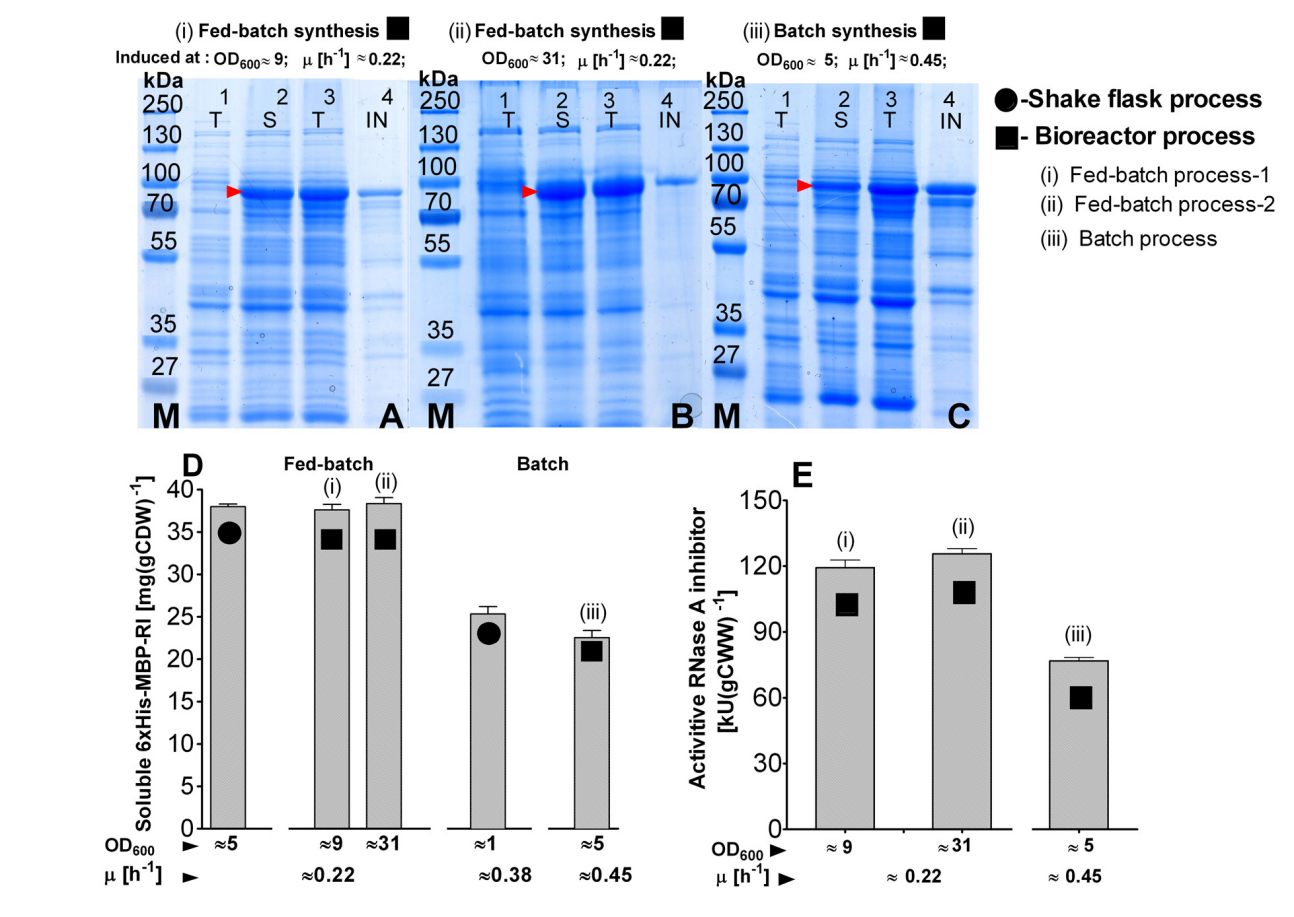
**SDS-PAGE (A-C) and derived product amounts (D) of samples from the expression of 6 × His-MBP-RI in *E. coli *RV308/p*ibpfxsT7lucA*/pCTUT7MBP-RI fed-batch and batch cultures**. (A) Bioreactor process-1, (B) Bioreactor fed-batch process-2, (C) Bioreactor batch process. Lanes: 1 - total protein fraction 10 min before induction, 2 to 4 - soluble protein fraction (2), total protein fraction (3) and insoluble protein fraction (4), all 4 hours of induction. (D) Yield of soluble 6 × His-MBP-RI in mg per gram of cell dry weight [mg (g CDW)^-1^] in relation to the specific growth rate at the time of induction. Lane abbreviations: T - total, S - soluble, IN - insoluble protein fractions. Lanes marked with M: protein size marker PageRuler™ Protein Ladder Plus from Fermentas Ltd. (E) Detected ribonuclase inhibitor activities (in kilo units) in the cellular crude extracts, calculated for 1 gram of cell wet weight [kU (gCWW)^-1^] after bioreactor batch and fed-batch cultivation with 6 × His-MBP-RI protein production at 37°C in *E. coli *RV308/p*ibpfxsT7lucA*/pCTUT7MBP-RI.

The amount of the soluble 6 × His-MBP-RI protein per cell unit at the different cell densities was nearly the same in both fed-batch bioreactor runs and also the same as in the EnBase^® ^shake flask with analog cultivation conditions (figure 9, SDS PAGE images A and B, charts D, E). The cellular productivity in the fed-batch bioreactor was maintained at the same efficiency level independently from the pre- and post induction cell densities (figure 9, images A, B and charts D, E). Thus the final volumetric product yield was higher in the fermentation where recombinant protein production was induced at the higher cell density.

The significance of specific growth and substrate uptake rates on the production of 6 × His-MBP-RI protein was also verified at the bioreactor scale by target protein production with the batch cultivation mode (figure 8C). All batch cultivation and target protein production parameters (i.e. medium composition, rate of aeration and pO_2 _level, cultivation temperature) were maintained the same as in the fed-batch production processes. In batch boreactor cultures 6 × His-MBP-RI protein synthesis was induced at an OD_600 _of approximately 5 (μ = 0.45 h^-1^) and the cultivation was continued for 4 hours at 37°C (figure 8C).

As earlier shown in the shake flask experiments, the batch production process resulted in a 40% lower production of soluble product, which was also confirmed with the activity test (figure 6). Interestingly, however the total amount of product was similar to the fed-batch process 1, but much more of the product was detected in the insoluble cell fraction. Compared to the corresponding shake flask culture, the batch bioreactor production process had a 10% higher total protein yield, but the soluble 6 × His-MBP-RI protein in bioreactor was 8% lower (figures 6, 7 and figure 9 charts D, E).

## Discussion

### The high throughput screening in 96 microwell plates

This study to our knowledge is the first documentation for an example of performing the development of a recombinant process from a library screening stage upwards to process fully straight by the fed-batch cultivation mode. The approach is very simple and fast. In our case all stages, including the expression library generation, could be performed in about a month's time.

Firstly, we created a new plasmid library, which is complementary to the earlier periplasmic library described by Kraft et al. [7]. The new library consists of 45 vectors which contains the three promoters of different strength, and the three ribosome binding sites from the old library, but the new plasmid set also contains 4 different N-terminal fusion partners, SUMO, thioredoxin (TRX), maltose binding protein (MBP), and glutathione S-transferase (GST). Additionally each vector contains an N-terminal 6 × His tag (see Results section, Figure 1). This library can be combined with the luminescence based monitoring plasmid for the heat shock like response which has been described before [8].

Secondly, we used this plasmid library to screen for soluble production of a heterologous protein, an RNase inhibitor (RI), which is highly aggregating during synthesis under standard conditions (own data, not shown), which also became evident by the library screening. The screening was performed from the beginning with a fed-batch like procedure which, very surprisingly and importantly, was shown for this protein to be important. The process was successfully scaled up from 96 microwell plates to the bioreactor scale.

By utilizing the EnBase^® ^substrate autodelivery system in 96 microwell plates, we implemented large-scale cultivation mode conditions already at the micro-scale high-throughput screening stage for the optimal RI fed-batch synthesis platform. In addition the systematic expression vector library in tandem with the monitoring system for the heat shock like response allowed to derive the key information about significant genetic features stipulating cytoplasmic accumulation of the tagged RI into the soluble fraction in very short time. The analyses of tagged RI expressed in the micro-scale cultures during the established fed-batch cultivations, showed a high robustness of this procedure towards the cultivation conditions, the cellular physiological states and the cellular synthesis capacity. Surprisingly, in contrast to our expectations, the variations in ribosome binding site and/or promoter did not significantly effect the cytoplasmic accumulation of soluble tagged RI. The only factor which yielded a higher solubility was the fusion with MBP, which was concluded to be an effective aid for RI cytoplasmic accumulation in the soluble state at the given expression conditions.

### Evaluation of cultivation factors for recombinant fed-batch production

Interestingly, in difference to many other examples described in the literature, in the RI case, the amount of soluble and active product was neither influenced by the cultivation temperature nor by induction strength which was here modulated by the promoter strength. In contrast medium composition and preinduction specific growth rate had a very drastic effect on the accumulation of soluble and/or total 6 × His-MBP-RI protein.

Based on the screening data the further process development was continued with the pCTUT7 expression vector, containing the strongest promoter and ribosome binding site versions, as well as 6 × His-MBP as fusion partner.

In the shake flask scale EnBase^® ^was applied to investigate the effect of the specific growth rate on the yield of soluble product. Therefore, simply IPTG was added at different times which reflect different specific growth rates. The highest amount of 6 × His-MBP-RI was obtained when induction was performed at a μ of 0.22 h^-1^. Interestingly, the amount of soluble 6 × His-MBP-RI protein was lower in shake flasks performed under batch conditions with different media, which may be due to the fast growth and corresponding high product accumulation rate leading to aggregation (see Result section, figures 6, 7). In the different EnBase^® ^cultures the lowest amount of the 6 × His-MBP-RI was obtained when cultures were induced after 14 hours of cultivation at the specific growth rate of 0.1 h^-1 ^at an OD_600 _of 11. This low yield was possible due to insufficient cellular physiological activity, thus cells were incapable to maintaining high level protein production (see Result section, figures 6, 7). The results are in good agreement with optimal preinduction growth rates detected by others, e.g. 0.2 h^-1 ^by Bentley et al. [18] and 0.27 h^-1 ^by Flickinger and Rouse [19] and well above the critical growth rates for growth under glucose limitation of approximately 0.15 h^-1 ^[19-21] where the yield of recombinant protein production is often seen to decrease due to increased maintenance requirements and starvation responses [22-24].

### Bioreactor Process

During the scale-up it was possible to maintain the cell specific production rate and, at the same time, to increase the volumetric yield by continuing the bioreactor cultivations to a higher cell density. By having information about optimal fed-batch cultivation conditions for efficient recombinant synthesis the fed-batch process was successfully reproduced even at higher cell densities in the bioreactor scale. According to the information extruded from the shake flask cultures MSM medium with a feeding mode stipulating a specific growth rate of μ = 0.22 h^-1 ^was considered as optimal for the bioreactor process. The 6 × His-MBP-RI production by the fed-batch mode in shake flasks and in the bioreactor showed that by tuning the optimal substrate uptake rate we created highly similar production conditions in the EnBase^® ^shake flasks to a stirred bioreactor. Indeed, the main difference between the fed-batch flask and bioreactor cultivation was only the higher oxygen transfer capacity in the bioreactor. Conseqently, higher cell densities could be reached, however, the specific production pattern (soluble to insoluble fraction) of the product was not influenced.

Batch cultivations in the bioreactor scale did not just allow to confirm that 6 × His-MBP-RI protein soluble accumulation is inversely related to the specific growth rate, but also indictate that the variation by scaling up from batch cultures is bigger than the variation during the scaling of a fed-batch process (see Results section, figure 9).

Our results confirm the importance of applying the fed-batch mode from the beginning of process development. Using different conditons during initial screening and later in the bioreactor scale leads to selection of conditions in the first stage which are not optimal or relevant for the second stage and would turn out in laborious optimisation steps. Currently more and more experimental appear which prove this. Clearly this conclusion is not related to *E. coli *recombinant cultures, but also has been extensively discussed in a recent paper by Scheidle et al. [25] at the example of a study with the yeast *Hansenula polymopha*. Also in this case the authors observed significant viariation of the expression of a target protein (GFP) in dependence o the cultivation conditons, and the fed-batch mode had a positive effect on the product yield.

The specific growth rate has been in many studies proven to be an important parameter for the accumulation of active and soluble product. Substrate delivery techniques like Enbase or the FeadBead system with an approximate constant rate of glucose release provide a good basis to evaluate this effect. It may be however remarked, that the volumetric yields in small scale shaken cultures are restricted by the relatively low oxygen transfer rate compared to bioreactors. However, different new approaches such as baffled microwell plates [26] or disposable mini-scale reactors with improved mixers [27,28] may also increase the yield in the small scale in future and become valuable tools to enhance the strength of screening procedures.

## Conclusions

In this work we demonstrated that the high-throughput screening approach based on fed-batch type cell cultures in shaken 96 microwell plates is a suitable tool for a fast, cheap, and systematic bioprocess development based on parallel evaluation of a large number of expression platforms at uniform cultivations conditions.

During the development of the recombinant fed-batch process, by employing the EnBase^® ^technology in 96 microwell plates and shake flasks, several important practical observations may be discussed. Firstly, as it was learned from later EnBase^® ^microwell plate cultivations (results not shown) the first culture synchronization step is not necessary - the cultures are perfectly synchronized after 16-24 hours fed-batch cultivation due to the controlled glucose release if a small amount of glucose is added initially. Indeed it would be even more beneficial for the synchronized cultivation if the stock culture is produced from culture suspension after the uniform cultivation in liquid medium. In our case, due to the large number of expression clones we prepared the glycerol stock cultures directly from the transformation plates. Thereby it was more risky that a direct culture synchronization without a preculture would succeed. However in a new variant, EnBase^®^-Flo [29], it should be possible to add the biocatalyst after an initial small batch phase, which would also for libraries make the preculture step obsolete.

The amount of material for analysis of protein synthesis in 96 microwell plates is quite low. Therefore, the samples were restricted to the point of induction and to the final point of cultivation, which also resulted in less disturbance of the culture, which would have been caused by regular sampling. Despit this we could show in this study that fed-batch cultivations in the 96 microwell plate and shake flask scale are very similar. As a intermediate scale of cultivation we now use widely 24 deepwell plates, which provides the benefits of parallel screening in combination with a larger cultivation volume wich allows more analyses and due to a lower impact of evaporation a more robust process.

Our results derived from batch and fed-batch processes in different scales clearly showed for the RNase inhibitor that cultivation factors, aside from the vector construct, have a key impact on the yield of soluble product. Interestingly, controling of the specific growth rate was much more important than regulating genetic factors - promoter strength and ribosome binding sites. It remains interesting to see whether this holds true also for other proteins.

In addition and most importantly, for the RNase inhibitor traditional approaches with the screening of the library with complex medium would have totally failed to select the right clones. The use of mineral salt medium in combination with the fed-batch technology turned out to be absolutely critical for our success to develop a robust process. We believe that our results may be very relevant and transferable to many other screening studies which are currently performed with complex media as a standard procedure and therefore may miss favourable clones.

## Methods

### Vector library preparation

The RI encoding gene was designed for insertion into an expression vector library via site specific recombination reaction based on the Gateway^® ^cloning technology by PCR end extension (with High Fidelity PCR mix from Fermentas) according Invitrogen's Gateway cloning manual (for details see: http://www.invitrogen.com). The PCR fragment was purified from 1% agarose with the QIAquick Gel Extraction Kit (Qiagen) and inserted into the pDONR201™ (Invitrogen) vector via Gateway^® ^cloning "BP recombination". The whole Gateway^® ^BP recombination reaction was transformed into the *ccdB *gene effect sensitive *E. coli *DH5α strain via calcium transformation. The halves of the transformation mixture were plated on Luria broth (LB) plates with 50 μg mL^-1 ^of kanamycin. The pDONR201™ vector, containing the target gene, was purified with the GeneJET™ Plasmid Miniprep Kit (Fermentas) and used for "Gateway^® ^LR" recombination for insertion of the target gene into to the destination protein expression library. The recombination mixture was transformed into the *ccdB *sensitive *E. coli *DH5α strain via calcium transformation and plated on LB solid medium with 30 μg mL^-1 ^of chloramphenicol. The expression vectors, containing the target gene, were purified with the GeneJET™ Plasmid Miniprep Kit (Fermentas) and used for the following transformations.

#### Preparation of target protein expression strain library

The expression strain *E. coli *K12 RV308 (ATCC 31608) was first transformed with the reporter plasmid *pibpfxsT7lucA *previously described by Kraft et. al. [8], carrying a resistance for ampicillin, and plated on LB agar with ampicillin (100 μg mL^-1^). The expression strain *E. coli *RV308 *pibpfxsT7lucA *was co-transformed with the RI gene containing the cytoplasmic expression library; the transformants were plated on LB agar containing ampicillin (100 μg mL^-1^) and chloramphenicol (30 μg mL^-1^). Both transformations were based on the calcium temperature shock method. The cell stock was produced by washing the transformants from the surface of the agar plates with 2 mL of glucose-based mineral salt medium (MSM), containing the antibiotics and glycerol (25%). The collected cell suspensions having cell densities (OD_600_) of about 10 to 30 were aliquoted into sterile PCR stripes and stored at -70°C.

### Cultivation media

Transformations and plasmid propagations were performed on solid and liquid LB medium containing Bacto-Tryptone (10 g L^-1^), Bacto-yeast extract (5 g L^-1^), NaCl (10 g L^-1^), 15 g L^-1 ^bacto agar (if solid medium) and the required antibiotics. Fed-batch and batch cultivations were performed in glucose-based mineral salt medium (MSM) with the following composition (per litre): Na_2_SO_4 _2 g, (NH_4_)_2_SO_4 _2.68 g, NH_4_Cl 0.5 g, KHPO_4 _14.6 g, NaH_2_PO_4 _× H_2_O 3.6 g, (NH_4_)_2_-H-citrate 1.0 g, and glucose 2.5 to 15 g. NaOH (40%) was used to adjust pH to 7.0 prior to the heat sterilisation. Semi-synthetic medium was based on the MSM with additional 10 g L^-1 ^of yeast extract and 10 g L^-1 ^casamino acids. Additionally, before cultivation on the mineral salt and semi-synthetic media the following sterile solutions were added: 2 mL L^-1 ^of (1 M) MgSO_4 _and 2 mL L^-1 ^of trace element solution with the following composition (per litre): CaCl_2 _× 2H_2_O 0.5 g, ZnSO_4 _× 7H_2_O 0.18 g, MnSO_4 _× H_2_O 0.1 g, Na_2_-EDTA 20.1 g, FeCl_3 _× 6H_2_O 16.7 g, CuSO_4 _× 5H_2_O 0.16 g, CoCl_2 _× 6H_2_O 0.18 g; as well as 100 μL L^-1 ^of thiamine hydrochloride (1 M), 1 mL L^-1 ^of ampicillin (100 mg mL^-1^) and 1 mL L^-1 ^of chroamphenicol (30 mg mL^-1^). The feeding solution for fed-batch cultivations was based on fully formulated MSM with the required antibiotics and 550 g L^-1 ^of glucose.

### Fed-batch mode cultures and recombinant protein synthesis in 96 microwell plates

EnBase^® ^technology (BioSilta Oy, Oulu, Finland) based fed-batch 96 microwell plate cultures were performed in 96 well flat bottom plates (Perkin Elmer). The wells of the plates were filled with heat sterilized gels, referred as - "bottom" and "top" phases [11]. Firstly, 100 μL of "bottom" gels (1.5% of Bacto-agar, Difco) and 10% of potato starch (Sigma) were added. After solidification of the bottom gels, 50 μL of top-gels (3.25% Bacto-agar) were added. The wells for cultivation were filled with 150 μL of fully formulated sterile MSM containing 100 μg mL^-1 ^of ampicillin and 30 μg mL^-1 ^of chloramphenicol. For cultivation the plates were closed with a plastic lid. All microscale pre-induction cultures were performed by intensive shaking with a Variomag^® ^Thermoshake (Inheco, Germany) at 37°C and 750 rpm (amplitude 1.5 mm). The inoculum plate with the gel-based EnBase^® ^system was inoculated with 5 μL of glycerol stock cultures per well. Synchronization of all 45 RI expression strain cultures was performed in these plates in the batch mode in MSM containing 2.5 g L^-1 ^of glucose at 37°C for 12 hours (overnight). After overnight cultivation at the obtained cell densities (OD_600 _about 4.5 ± 1.0) the release of glucose from starch was started by addition of 5 μL of amylase (BioSilta Oy, Oulu, Finland) to obtain a final concentration of 6 AGU L^-1 ^(amyloglucosidase units per liter). 1 AGU is the amount of enzyme which releases 1 μmol min^-1 ^of maltose).

Additionally the culture suspensions were supplemented with 5 μL of NH_4_OH (25%). The inoculum cultures were cultivated for another 12 hours under glucose limitation at 37°C. After the initial cultivation 5 μL of synchronized and adapted precultures were transferred to the gel-containing wells of a new microwell plate (with 6 AGU L^-1^), from the beginning possessing linear glucose auto-release and cultivated for 9 hours at 37°C. Target gene expression was induced after 9 hours of cultivation at OD_600 _= 12 ± 0.5 by addition of 5 μL of IPTG, dissolved in fully formulated medium, to achieve a final concentration of 0.5 mM. At the time of induction also the cultivation temperature was shifted to either 37°C, 30°C, or 22°C, respectively. In all experiments the cultures were harvested 7 h after induction.

### Determination of cell growth in microwell plates

The initial (0 h time point) optical density (OD) of all microwell plate cultivation samples, was determined by measuring the turbidity with a Victor^3 ^plate reader (PerkinElmer) with the following settings: wave length 490 nm, 15-20 sec shaking before plate reading. Cell densities from EnBase^® ^cultures were determined by 30 fold dilution of 5 μL broth samples in clear deionized water or cultivation medium in a final volume of 150 μL in clear, flat bottom 96 microwell plates (PerkinElmer). The OD_490 _obtained by using the Victor^3 ^were recalculated to OD_600 _with a 1 cm path length using the following equation, obtained by a calibration curve:

where D_f _is the dilution factor.

### Fed-batch mode cultivations in shake flasks

The EnBase^® ^technology based fed-batch shake flask cultivations were performed in 1 L baffled Erlenmeyer flasks in 200 mL of MSM. These shake flasks contained 100 mL of the "Bottom gel" (10% potato starch, 5% Bacto-agar) and 75 mL of the "Top gel" (5% Bacto-agar). The inoculum for the production cultures was prepared by overnight batch cultivation at 37°C in 100 mL of MSM containing 7 g L^-1 ^of glucose and the appropriate antibiotics. For inoculation the cultures were washed and resuspended in 50 mL of fully formulated sterile MSM without glucose. 10-12% from the total cultivation volume of culture suspension was transferred to MSM EnBase^® ^cultivation flask to achieve final volume of 200 mL, with the appropriate antibiotics and no glucose. 1 mL of glucoamylase, diluted in sterile deionized water, was added to the cultivation medium, just after inoculation to obtain a final amylase concentration of 12 AGU L^-1^. The cultivations were performed at 37°C and 180 rpm on a Multitron shaker with an orbit of 2.5 cm (Infors). Product - 6 × His-MBP-RI synthesis was induced at three different time points (t_1 _to t_3_) which corresponded to the following specific growth rates: t_1 _= 4 h, OD_600 _= 2.0 ± 0.2, μ_1 _≈ 0.33 h^-1^; t_2 _= 8 h, OD_600 _= 5.5 ± 0.2, μ_2 _≈ 0.22 h^-1^; and t_3 _= 13 h, OD_600 _= 11 ± 0.2, μ_3 _≈ 0.1 h^-1^. Induction was performed by addition of IPTG (1 M) to achieve a final concentration of 0.5 mM. The protein was produced for 4 h, at 37°C, shaking at 180 rpm.

### Batch mode cultivations in the shake flasks

The inoculums for batch protein production in the shake flasks were prepared by overnight batch cultivation of the selected clone in 100 mL volume shake flask with 10 ml of LB at 37°C. For protein production, 1% of the corresponding inoculum culture was transferred either to fresh LB, or to mineral salt medium, or to a semi synthetic medium, both containing 10 g L^-1 ^of glucose, to a final volume of 200 mL in 1 L baffled Erlenmeyer shake flasks. Cultures were cultivated at 37°C and 180 rpm until they reached the induction point, corresponding to a cell density of OD_600 _= 1 ± 0.05. Induction was performed by addition of IPTG (1 M) to a final concentration of 0.5 mM. The MBP-RI fusion was synthesized for 4 h, at 37°C.

### Bioreactor processes

Batch and Fed-batch cultivations were performed in a 10 L working volume Biostat C bioreactor (with MFCS/win 2.0 supervisory system, B. Braun Biotech, Melsungen, Germany) with the following parameters: the pO_2 _was maintained at 30% by adapting the stirrer rate and automatic regulation of the air flow (from 0 to 30 liters per min), the cultivation temperature was 37°C (before and after induction), pH was controlled at 7.0 ± 0.1 by addition of NH_4_OH (25%) or H_3_PO_4 _(2 M). MSM containing 15 g L^-1 ^of glucose was used in the batch production process.

The fed-batch cultivations were started with a volume of 8.5 L of MSM, and contained 4.5 g L^-1 ^and 15 g L^-1 ^of glucose, respectively. The feeding was controlled by the Biostat software (version 4.62).

Exponential feeding profiles were programmed to maintain a specific growth rate of μ ≈ 0.22 h^-1^. The feeding profiles were calculated with the following equations:

where *F*_*o *_is the initial feeding rate [L h^-1^], *μ *is the specific growth rate [h^-1^] to be maintained during feed operation, and *t *is the time after feed start [h]. The initial feeding rate was calculated from the mass balance on substrate according to.

Here, *X*_*0 *_and *V*_*0 *_are the cell dry weight (CDW) [g L^-1^] and the culture volume [L] at the time of the feeding start, respectively, *S*_*f *_[g L^-1^] is the substrate concentration in the feeding solution, and Y_x/s _is the yield coefficient (g CDW per g of glucose). *Y*_*x/s *_in all calculations was 0.3 g g^-1 ^as calculated from batch fermentations.

Before initiation of the fed-batch mode cells were cultivated as a batch until OD_600 _≈ 6 or ≈ 22, respectively. The biosynthesis of the product was induced during the fed-batch mode at OD_600 _≈ 9 or OD_600 _≈ 31, respectively. The specific growth rate at the time of induction was the same in both cases, 0.22 h^-1^. After induction by 1 M IPTG cultivations were continued for 4 h at 37°C under continuation of the exponential feed function. In the batch process, performed as a control, 6 × His-MBP-RI target protein synthesis was induced at OD_600 _≈ 5 (μ ≈ 0.45 h^-1^) and the culture was continued for 4 hours at 37°C.

### Luciferase assay-target protein misfolding stress monitoring

After OD_600 _determination the analyzed bacterial cultures were separated from the cultivation medium by centrifugation in a microcentifuge (14000 rpm, 5 min). The cell pellet was resuspended in 0.9% NaCl and diluted to achieve 8 × 10^7 ^cells mL^-1 ^(corresponding to 0.10 OD_600_) in a final suspension volume of 200 μL. The whole 200 μL suspensions were transferred to the wells of a 96 microwell plate (Greiner) with transparent bottom. Then 100 μL of fresh reaction buffer (25 mM tricine, 15 mM MgCl_2_, 5 mM ATP, 7 mM beta-mercaptoethanol, 0.5 mg mL^-1 ^bovine serum albumin, 13 mM D-luciferin Na-salt, pH 7.8) were added and the luminescence was measured with a Victor^3 ^multilable counter (Wallac) every 10 min at 25°C over a total time of 60 min. Blank samples were included (expression strain cultures cells cultivated without induction). The "true" luminescence values were calculated for each sample from the average of the measured values during the plateau phase by applying the formula:

where Tv - True, Mv - Measured and Bv - Blank luminescence values calculated by formula Bv = (Blank signal) × Df × N, Df - dilution factor and N - normalization factor for the cells amount corresponding to cell density of 1 at OD_600_.

### Protein analysis

For visualization and quantification of the soluble and insoluble protein fractions from microwell plate cultures, normalised amounts of cellular suspension were transferred to 1.5 mL Eppendorf tubes, harvested by centrifugation (10 min, 14000 rpm, 4°C) and the pellet was resuspended in 100 μL of lysis buffer (50 mM Tris-HCl pH 8.0, 0.1% Triton X-100, 1 mM EDTA, 1 mM PMSF, 5 mM DTT, 0.1 mg mL^-1 ^lysozyme). The whole cellular suspensions were sonicated for 10 sec with a Vibra cell™ sonicator (Sonic and Materials Inc., 2 mm diameter probe tip) at 4°C. The supernatant (soluble fraction) was collected after centrifugation (10 min, 14000 rpm, 4°C) and the pellet (insoluble protein fraction) was resuspended in 100 μL of lysis buffer without lysozyme. Samples for SDS-PAGE were prepared as follows: 25 μL of 5× SDS-PAGE loading buffer, 5 μL of 20 × DTT (Fermentas Ltd.) and 20 μL of deionized water were added to 50 μL of the respective protein suspensions in order to obtain a final sample volume of 100 μL. Samples were heated for 15 min at 95°C. 10 μL of sample was applied to each lane of a 10% SDS-PAGE gel.

Cell samples harvested from flask and bioreactor cultivations were resuspended in lysis buffer with the following ratio: 1 g of biomass with 5 mL of lysis buffer. Lysis was performed by sonication for 30 sec (Vibra cell™, Sonic and Materials Inc., 6 mm diameter probe tip) at 4°C. The lysate was distributed to 1 mL fractions and centrifuged (10 min, 14000 rpm, 4°C). The supernatant (soluble protein fraction) was collected and the insoluble protein fraction containing pellets from the 1 mL disrupted cell suspensions were resuspended as described above in 1 mL lysis buffer without lysozyme. 10 μL of soluble protein and cellular debris suspensions were taken for SDS-PAGE sample preparation. Final volumes of 100 μL of SDS-PAGE samples were obtained as described above and 10 μL of these suspensions were applied for the SDS-PAGE run.

Quantification of the target protein in soluble protein fractions was performed after separation on an Agilent 2100 bioanalyzer. Therefore the normalised crude extracts were 4-fold diluted in the buffer containing 50 mM of Tris-phosphate pH 8.0 and 1 mM EDTA. Evaluation of protein amounts on SDS-PAGE gels was performed by using TotalLab software (Total Lab Systems). The ribonuclease inhibitor activity in the normalised for the biomass quantity crude extract was determined by activity assay described by Blackburn et al. [17].

## Competing interests

The authors declare that they have no competing interests.

## Authors' contributions

JS designed the experimental setup, performed all cultivation experiments and prepared the manuscript. JPP helped with the screening set-up and RR contributed to the bioreactor experiments and product analysis. MK and UH constructed the cytoplasmic expression library. PN initiated the project, assisted with data analysis and manuscript preparation. All authors read and approved the final manuscript.

## Supplementary Material

Additional file 1 **Supplementary figure 1**. Aggregation signal measured as luminescence in samples of the RI expression library consisting of 45 different expression vectors propagated in *E. coli *RV309 p*ibpfxsT7lucA *in 96 microwell plates by the EnBase^® ^technology 7 hours after induction for the cultures performed at 30°C (A) and 22°C (B). Bars are numbered in respect to the expression system: 1 - pCT7, 2 - pClac, 3 - pCVar, 4- pCUT7, 5 - pCUlac, 6 - pCUvar, 7-pCTUT7, 8-pCTUlac, 9- CTUVar. Click here for file
