# Supplementary figures and images for: Novel approach of high cell density recombinant bioprocess development: Optimisation and scale-up from microlitre to pilot scales while maintaining the fed-batch cultivation mode of E. coli cultures
Source: Microb Cell Fact. 2010 May 20;9:35. doi: 10.1186/1475-2859-9-35 (PMC2890543; doi:10.1186/1475-2859-9-35)

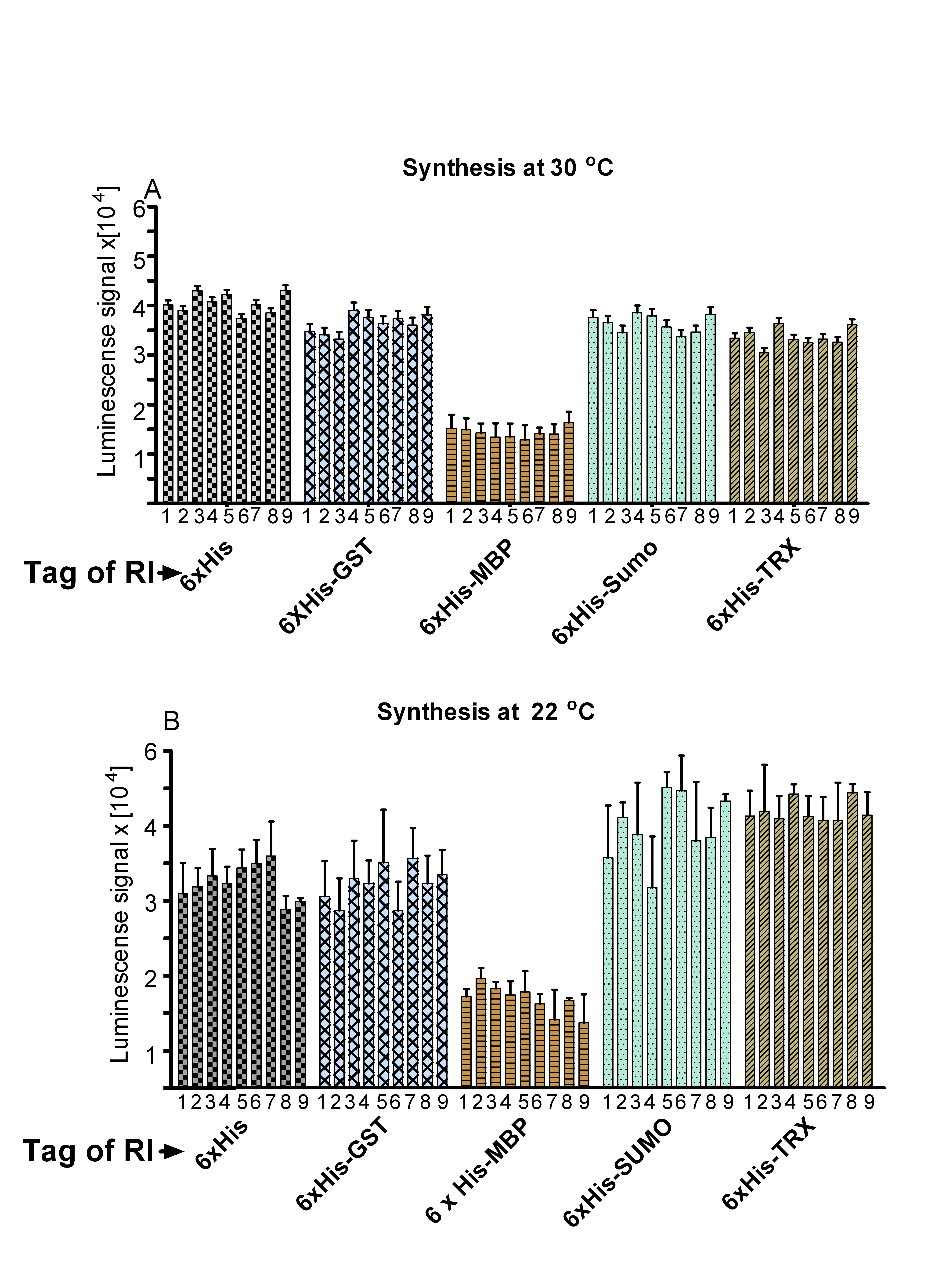

Supplement: Additional file 1 — Supplementary figure 1. Aggregation signal measured as luminescence in samples of the RI expression library consisting of 45 different expression vectors propagated in E. coli RV309 pibpfxsT7lucA in 96 microwell plates by the EnBase® technology 7 hours after induction for the cultures performed at 30°C (A) and 22°C (B). Bars are numbered in respect to the expression system: 1 - pCT7, 2 - pClac, 3 - pCVar, 4- pCUT7, 5 - pCUlac, 6 - pCUvar, 7-pCTUT7, 8-pCTUlac, 9- CTUVar. [file 1475-2859-9-35-S1.JPEG]
